# Modelling collaboration of primary and secondary care for children with complex care needs: long-term ventilation as an example

**DOI:** 10.1007/s00431-019-03367-y

**Published:** 2019-04-02

**Authors:** Daniela Luzi, Fabrizio Pecoraro, Oscar Tamburis, Miriam O’Shea, Philip Larkin, Jay Berry, Maria Brenner

**Affiliations:** 1 National Research Council, Institute for Research on Population and Social Policies, via Palestro, 32 – 00185, Rome, Italy; 2 School of Nursing & Midwifery, Trinity College Dublin, Dublin, Ireland; 3 Centre Hospitalier Universitaire Vaudois, Faculté de Biologie et de Médecine, Institut Universitaire de Formation et de Recherche en Soins, University of Lausanne, Lausanne, Switzerland; 4 Boston Children’s Hospital, Boston, USA

**Keywords:** Process modelling, UML, Professional collaboration, LTV, Multidisciplinary team

## Abstract

Children dependent on long-term ventilation need the planning, provision and monitoring of complex services generally provided at home by professionals belonging to different care settings. The collaboration among professionals improves the efficiency and the continuity of care especially when treating children with complex care needs. In this paper, the Unified Modelling Language (UML) has been adopted to detect the variety of the patterns of collaboration as well as to represent and compare the different processes of care across the 30 EU/EEA countries of the MOCHA project.

*Conclusion*: Half of the analysed countries have a multidisciplinary team with different degrees of team composition, influencing organisational features such as the development of the personalised plan as well as the provision of preventive and curative services. This approach provides indications on the efficiency in performing and organising the delivery of care in terms of family involvement, interactions among professionals and availability of ICT.

**Table:** 

**What is known:** • *Children with CCNs require a coordination of efforts before and after discharge in a continuum of care delivery dependent on the level of integrated care solutions adopted at country level.*	
**What is new:** •*The adoption of a business process method contributes to perform a cross-country analysis highlighting the variability of team composition and its influence on the delivery of care.* • *This approach provides indications on the efficiency in performing and organising the delivery of care in terms of family involvement, interactions among professionals and availability of ICT.*

## Introduction

Children with complex care needs (CCNs) have to access multiple points of care, not only to treat their conditions but also to monitor their psychophysical development through routine screening and examination tests. Moreover, given the complexity of the caring process, physical and emotional needs of parents and relatives have also to be considered [1–3] along with their active participation in the development of personalised care plans. The collaboration within and among care settings and professional specialisations is crucial considering that these children, when stable, are discharged to home. This requires a coordination of efforts before and after discharge in a continuum of preventive, curative and rehabilitation care delivery dependent on the level of integrated care solutions adopted at country level.

One of the main objectives of the MOCHA (Models of Child Health Appraised) project^1^ is to analyse the current approach in 30 EU/EEA countries to manage the care of CCNs, with particular regard to the integration of care among the primary, secondary and social level. This challenging task is carried out through a multidisciplinary perspective using different methods that complement each other in order to gain the most accurate picture possible of the variety of pathways adopted at country level for the care of these children. This has to take into account the different models of health systems in the European countries as well as the diverse ways of structuring, organising and addressing a child’s CCNs. Our contribution is to adopt a business process analysis [4] to capture patterns of care delivery in the highly dynamic, complex and multidisciplinary nature of health care processes [5]. This methodology is increasingly used also in health care [6–8] to explore two related aspects: (1) the control flow that specifies the activities performed as well as their order of execution to describe the process behaviour and (2) the organisational view that captures the professionals involved in the performance of each activity, highlighting their roles within the process of child’s care as well as their level of collaboration. This description helps analysing of the level of integration in the different parts of the process as well as identifying how this integration is achieved (e.g. multidisciplinary teams, co-location in the same facility, shared information systems, periodical communication). Moreover, the adoption of a standard language to describe the process (Unified Modelling Language, UML) in complex systems such as health care [9–11] supports a cohesive and pictorial description of the different ways of organising, coordinating and delivering child’s care, facilitating at the same time the comparison of the different patterns of care across countries.

This paper is focused on children dependent on long-term ventilation (LTV) due to chronic lung disease diagnosed at birth. LTV is a complex condition, generally managed, when stable, in the home environment. It affects an increasing number of children as a result of the progress in perinatal technology [12, 13]. The process description is based on a MOCHA questionnaire [14] that was developed for use with a scenario described in a vignette [15]. Questions were adapted from the *Standards for Systems of Care for Children and Youth with Special Health Care Needs* (CYSCHN) [16] and included a Complex Care European Survey of Change, adapted from the Eurobarometer Survey [17].

The paper is structured as follows: a methodology to describe the level of collaboration among professionals is presented, based on the UML use case and activity diagrams, as part of the framework proposed in [18]. It is then applied on the LTV case study identifying the different patterns of collaboration among the MOCHA countries. Finally, the results are discussed and conclusions are presented.

## Materials and methods

### Children assisted with long-term ventilation

The number of children assisted with long-term ventilation has consistently increased over the last 25 years. For example, some areas of the UK had a 30-fold increase in prevalence between 1994 and 2010, from 0.2 to 6.7 per 100,000 children [19]. This is consistent with other data available across the EU, with data from Italy showing a prevalence of 4.2 per 100,000 paediatric patients [20], and a report from Austria showing a prevalence of 7.4 per 100,000 [21]. This is supported by trends reported internationally. The number of children receiving long-term mechanical ventilation at home in Canada increased from 2 in 1991 to 156 children as of December 2011, with a twofold increase in the number of invasive ventilation initiations in the second 10 years, *n* = 45 (2001–2011) as compared to the first 10 years, *n* = 21 (1991–2000) [22]. Data from studies that have investigated long-term outcomes and quality of life for such children are scarce, but indicate reduced long-term health-related quality of life [23]. There is evidence of burn-out in parents within a short period of time of becoming the child’s primary care giver, with a recognised increase in physical or mental ill health issues, and a significant impact on family functioning [3]. Even though there are difficulties in providing appropriate mechanical ventilators for children and its management requires well trained care givers, many studies have shown that properly selected infants and children could be safely ventilated at home [24, 25]. The key factor of success is, however, the presence of a good team with dedicated members [24].

Following the MOCHA approach to fulfil the gap of data availability at European level, a set of ad hoc questionnaires sent to local experts in child health services (Country Agents (CAs)) were developed [14] to explore organisational and clinical aspects considering different tracer conditions at various children’s life stages. Some of them were specifically focused on children with CCNs to capture, in particular, how each EU/EEA country manages the interface between primary, secondary and social care. This is evident in the selection of questions that require information on the different parts of the child’s journey also including routinely health care service provision, such as preventive care. Each questionnaire is based on a vignette that captured the real-life context [26] and included sociocultural issues that can influence the family and care provision. As shown in the LTV vignette (Table 1), this approach allows to capture information on the basis of a non-experimental descriptive study and to perform both quantitative and qualitative analysis in the exploration of structures and processes of care provision for children [14]. This also allows to gain insights on the different levels of national management and treatment of children with CCNs providing a comparison across countries as well as across complex health conditions.

**Table. 1:** Vignette on LTV

Max is an 18-month-old boy with a diagnosis of chronic lung disease due to bronchopulmonary dysplasia. Max was born at 26 weeks’ gestation, weighing less than 1 kg. He had a diaphragmatic hernia, a gastrostomy tube placement at 3 months of age, and a grade 4 intraventricular haemorrhage requiring a cerebrospinal fluid ventricular shunt. Max has been dependent on a ventilator since he was born and is considered to have a life-threatening condition. A tracheostomy tube was placed at 6 weeks of age because of the need for ongoing ventilation. Max spent the first 3 months of his life in intensive care, followed by 4 months in a step-down transitional care unit. At present, Max has impaired pulmonary function, developmental delay in fine and gross motor skills, and speech and language difficulties. His prognosis for weaning off the ventilator does not seem favourable at the moment, and ideally, he requires the health-care input of the following health-care professionals: community nurses, specialist consultants (respiratory specialists, paediatricians, and neurologists), a community general practitioner, a pharmacist, a speech and language therapist, a physiotherapist, an occupational therapist, a social worker, a dentist, a home care nursing team, and respite care services. He lives with his two sisters, aged 5 and 7 years, and his mother and father. He lives 120 km from the main children’s hospital and 40 km from his nearest regional hospital, which has a small paediatric unit.	

### Data source

The level of collaboration among professionals was defined by analysing the answers provided by local CAs that collected comparative information across the 30 MOCHA countries to identify the different types of professionals involved in the performance of the child’s care activities. Answers from the CAs were also useful to understand the level of collaboration among settings, highlighting whether care professionals were part of a multidisciplinary team (MDT) belonging to a hospital and/or a primary care setting or individuals, each one carrying out specific professional-related activities. The questions adapted from the *Standards for Systems of Care for Children and Youth with Special Health Care Needs* (CYSCHN) [16] supported the identification of sub-processes that trace the care journey of children with CCNs, in particular:

1. Development of a written personalised plan that included the identification of the type of health services to be scheduled and provided after discharge as well as its implementation to monitor the provision of services and the child health status. The involvement of the family and/or the members of the health care team in writing the plan was also analysed as an indicator of care integration that specifically takes into account the family needs.
2. Organisation of the transition from the hospital to the child’s home or in a community-based setting analysing whether this service was provided by a hospital discharge planning coordinator. The presence of this type of professional also indicates another important feature of integrated care. In fact, the role of this professional is generally to advise and support the family establishing not only the appropriate equipment to be provided but also determining and scheduling the services in a home setting when the child is discharged [26].
3. Provision of general health services in the community distinguishing between basic services provided to all children independently from the complex condition and those specifically devoted to LTV issue.
4. Provision of preventative care screening and developmental checks. Additionally, in this case, the analysis distinguished between basic services provided to all children independently from the complex condition and those specifically devoted to LTV issue outlining practices of primary-secondary interface.

Moreover, the access to specialised services was investigated to capture the interface between primary and hospital care focusing the attention on the following activities:

- Access to psychological support targeted at child’s family including the professional who starts the process
- The direct access to a paediatric intensive care unit (PICU) in case of acute care treatment
- The management of palliative care

Within this process, the communication procedures adopted in each country to exchange information among professionals and settings were analysed as an important indicator of the level of care coordination in complex childcare management, where home care plays an important role.

### Business process modelling using UML

To capture the level of collaboration in place in each country in the performance of the care process, the professionals involved in each activity were gathered and classified on the basis of the following categories:

- A single health care professional that can either belong to primary care (e.g. paediatrician, community nurse, General Practitioner) or hospital (i.e. pneumologist, respiratory physiotherapist, paediatric cardiologist, paediatric surgeon);
- Multiple professionals not working in a team, that is individual professionals in charge of their specialty;
- MDT comprising hospital and/or primary care professionals;
- MDT that also comprises a social worker.

The result of this analysis was subsequently described using the UML model as part of the framework proposed in [18] that facilitates the comparison between the different business processes performed in the MOCHA countries. In particular, two UML types of diagrams were selected as suitable means to describe the interaction between the different stakeholders as well as the activities performed in each identified scenario [27].

The *UML use case diagram* described the process as an ideal set of macro-activities to be performed providing a country-independent picture. For each activity, it highlighted the type of professionals involved. Moreover, the use of the composition relationships (depicted by the symbol ) outlined the configuration of the team associated with the settings they belong to (primary, secondary and social service). This interaction was also shown using green notes that were associated to the type of actor that performed the related activity in each country. On the basis of this diagram, countries that had similar procedures, used similar services, and were based on similar caregivers for the provision of care were grouped, and a set of activity diagrams were modelled.

The *UML activity diagrams* clearly represented how each country/group of countries carried out the care process capturing the activities performed and the messages exchanged by the different actors as well as triggering conditions taking also into account the location and timeline of each activity. Within the process flow interrupting connector represented by a zig-zag arrow () was used to manage an exception that can occur during the execution of a relevant activity. Furthermore, this diagram adopted swim lanes for partitioning activities based on the actor that performs them. This partition was also useful to capture the interaction among actors and highlight the messages exchanged during the business process. In the paper, this partition was represented by vertical swim lanes. In our methodology, we adapted the UML convention adding a horizontal partition of the model to capture the different phases of the business process. It helped capturing the main sub-processes that constituted the whole process in line with the components of the CYSCHN [16].

A comparison of the activity diagrams was performed to detect differences and similarities among countries in the performance of the different parts of the care process also pointing out strength and weak points of each health system. This comparison can be also quantified using business process metrics [28, 29] that use the elements of the UML diagrams to assess the efficiency of the business process under investigation considering both the control flow (e.g. number of activities performed, the number of decision points, the hourglass within the process and the relevant waiting time, the information shared among professionals) and the organisational perspective (e.g. composition of teams, presence of systems adopted to exchange data and documents, role of the family within the process). Considering the purpose of this paper and giving the high-level description captured from the questionnaire, the analysis privileged the straightforwardness versus the fragmentation of the care process. Moreover, particular attention was given to the referral procedures related to the exchange of the personalised plan as an indicator of clinical integration.

## Results

The results reported in this section were based on the 23 CAs (77% of MOCHA countries) that provided applicable answers to the questions related to the management of the plan, the presence of discharge planning coordinator and the provision of preventive as well as general and specialist curative services.

### UML use case diagram

**Fig. 1:**
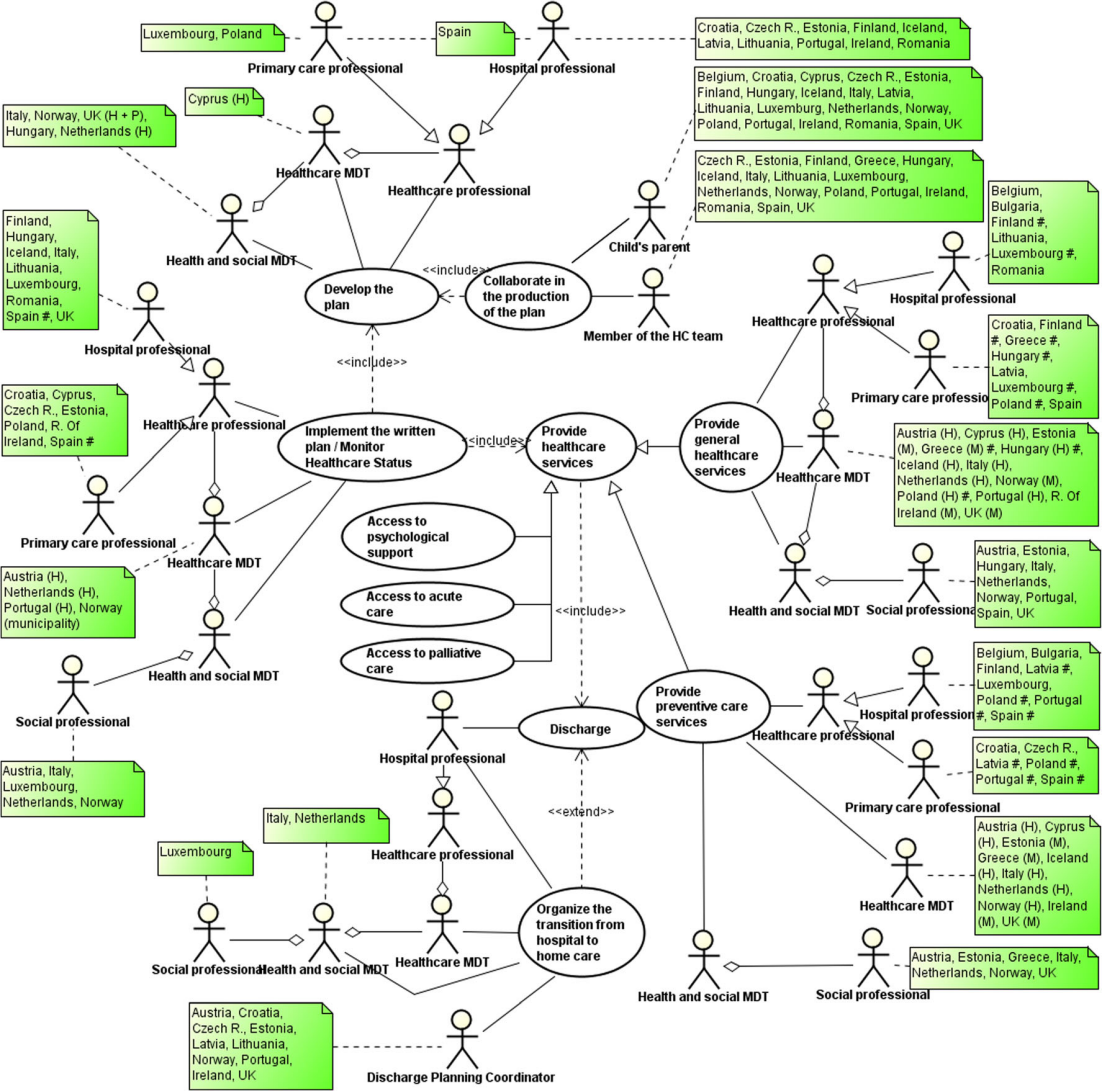
UML use case diagram. H = hospital, P = primary care; number sign = countries where the activity is performed by different actors

The UML use case diagram reported in Fig. 1 shows the macro-activities performed by health and social care professionals before and after discharge, highlighting the high variability of team composition across countries. The use case *Discharge* may comprise the *Organisation of the transition to home* as shown by the *extend* relationship between the two use cases. As reported in the diagram, ten CAs indicate the presence of a discharge planning coordinator (DPC). Among them, Croatia and Estonia specified that this role is covered by a nurse. In Italy and in the Netherlands, this task is carried out by a MDT, while in Luxemburg, a social worker may, if needed, cover this role. Considering the development of the plan, this task is performed in the hospital setting either by a single professional (e.g. Czech R. and Iceland) or by a MDT (e.g. Cyprus and the Netherlands). In Luxembourg and in Poland, this activity is performed by the primary care physician. In Italy, this is done in collaboration with territorial services. Moreover, the plan can be developed in collaboration with child’s parents and/or members of the team. This is highlighted by the *include* relationship between the use cases *Collaborate in the production of the plan* and *Develop of the plan*. Once the child is discharged at home, health and social care professionals are mainly involved in the implementation of the plan as well as in the provision of general and screening services as identified in the plan. These use cases *include* the *Discharge* use case as a generalisation of *Provide health care service*. In the diagram, additional use cases are added to represent the care coordination in case of medical crisis (acute care), and community-based service and support (palliative care and psychological support to parents) providing information on the procedures facilitating the access to care and/or the different actors starting the process. These specific activities are further investigated in the following paragraphs highlighting the professionals involved in their management as well as the composition of the relevant team, if present.

Within the high variability of the health care settings and professionals providing care, the use case diagram also shows different patterns of collaboration depending on the type of activity performed. Moreover, it is possible to single out countries in which all types of macro-activities after discharge are performed under the supervision of hospital professionals. This is the case of Austria, Iceland and the Netherlands that are organised in a MDT, while in Belgium, Bulgaria, Lithuania and Romania, a single health care hospital professional provides these services. Primary care services monitor the child’s care after discharge, providing both prevention and curative services in Croatia. In other countries (Estonia and Ireland), both the provision of preventive screening and curative services are carried out via collaboration between primary and secondary care. In Norway and in Hungary, this collaboration is in place in the provision of curative services, while in Spain, this is managed through the provision of preventive screening. In Finland and Greece, general services are provided within primary care, while hospital professionals are accountable for the specialised ones. In the UK, there is a close collaboration between hospital and community professionals working in a MDT that also includes a social worker.

### UML activity diagrams

Starting from the UML use case diagram, countries have been classified on the basis of two features: (1) the presence or absence of MDTs for the care of children on LTV and (2) the type and composition of health care organisations in charge of the child’s care. These features have been adopted to determine the extremes of a continuum of care where the lowest degree of integration occurs when there is a high fragmentation of service provision carried out by individual professionals. On the other extreme, a full integration is achieved when hospital and primary care professionals manage the whole child pathway in a MDT. Between these extremes, we have identified two additional patterns of collaboration: (1) MDT composed by hospital professionals and (2) a composition of two different MDTs respectively based on hospital and primary care settings. Moreover, the asterisks indicate the presence of a social worker. A similar analysis [30] has been used to measure the integration of care in a single country.

**Fig. 2:**
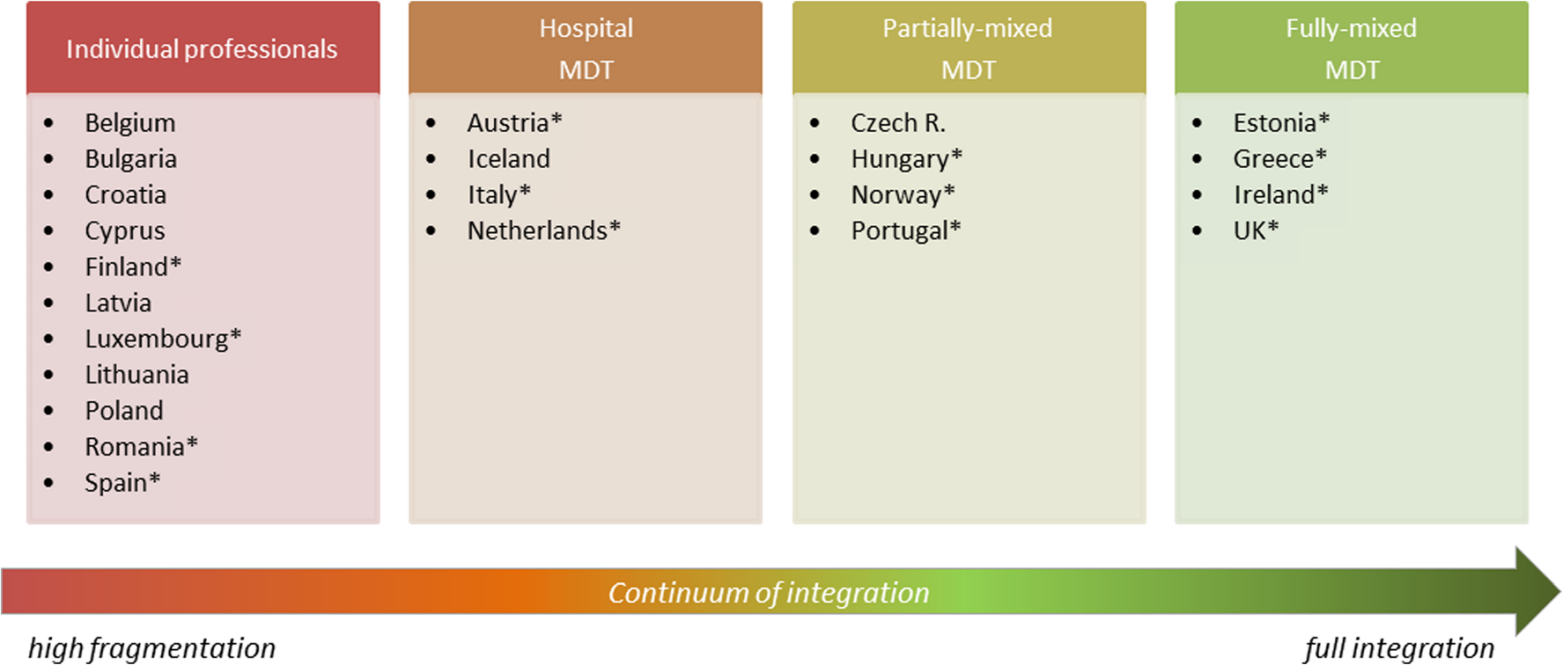
Clustering of countries within a continuum of integration

**Fig. 3:**
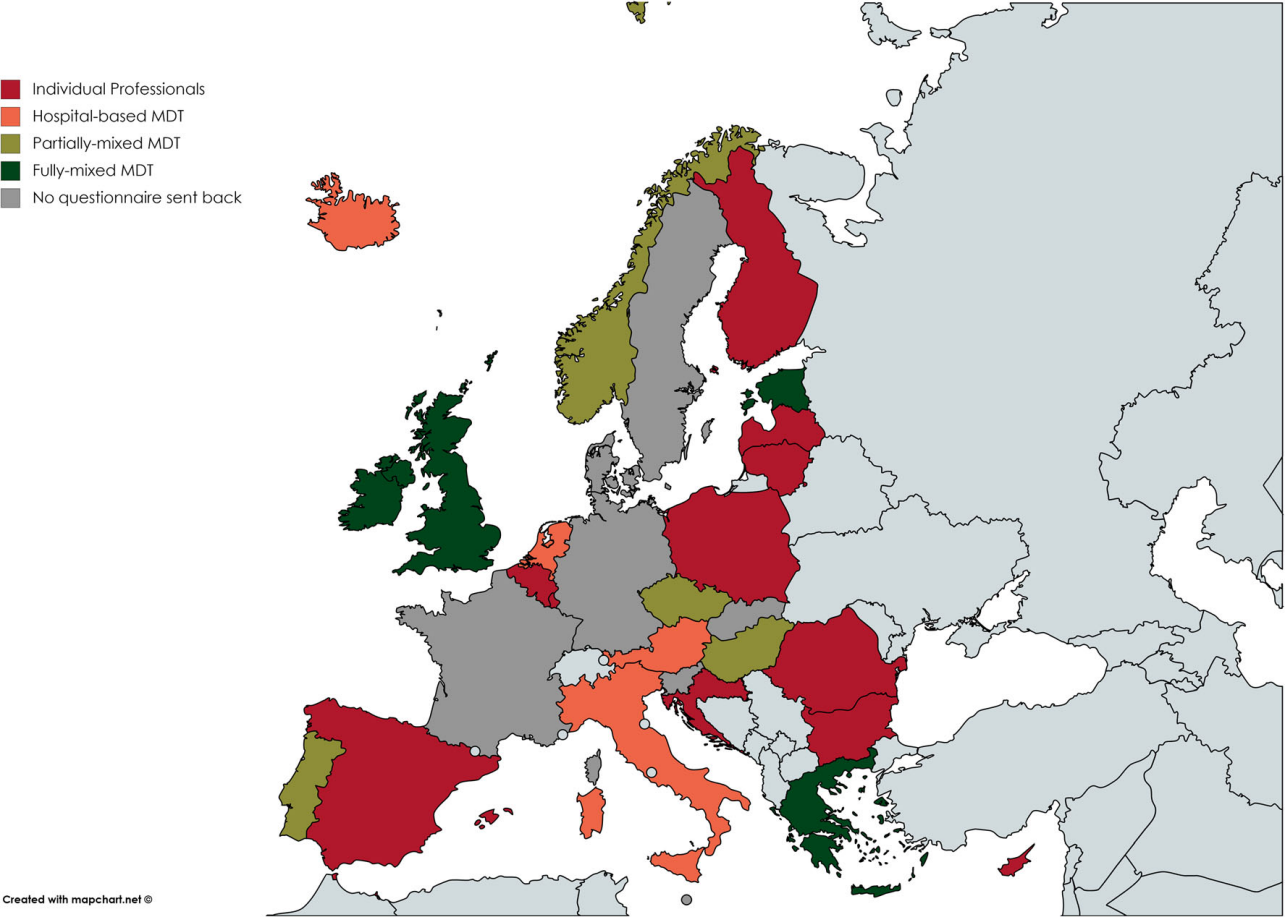
Classification of each MOCHA country in the relevant pattern of collaboration

Starting from this classification, the MOCHA countries have been clustered as shown in Fig. 2 and in the map reported in Fig. 3. Half of the analysed countries (*n* = 12) have a multidisciplinary team with different degrees of team composition, while in the rest of the countries (*n* = 11), care services are provided by individual professionals.

**Fig. 4:**
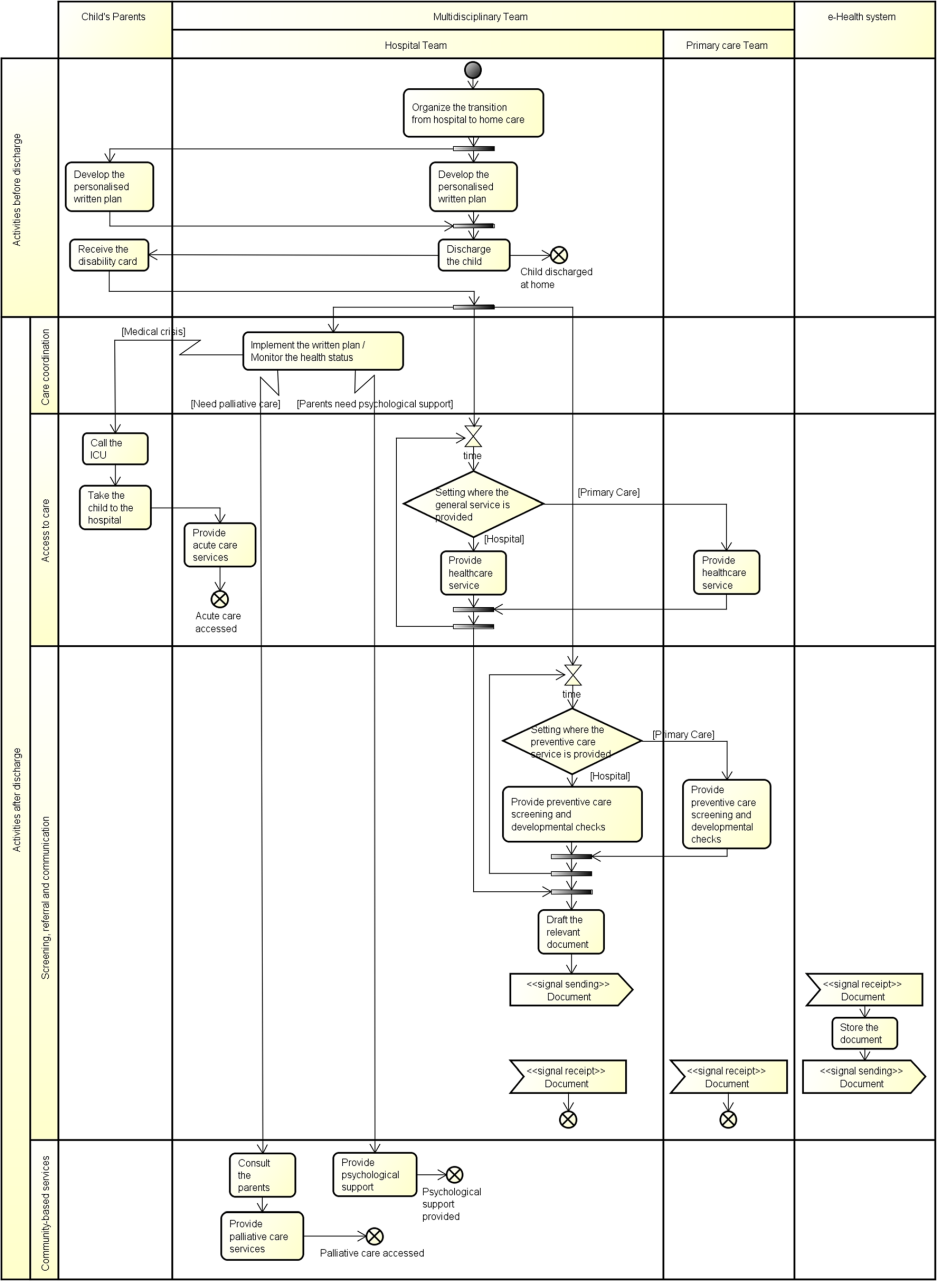
UML activity diagram: Estonia

**Fig. 5:**
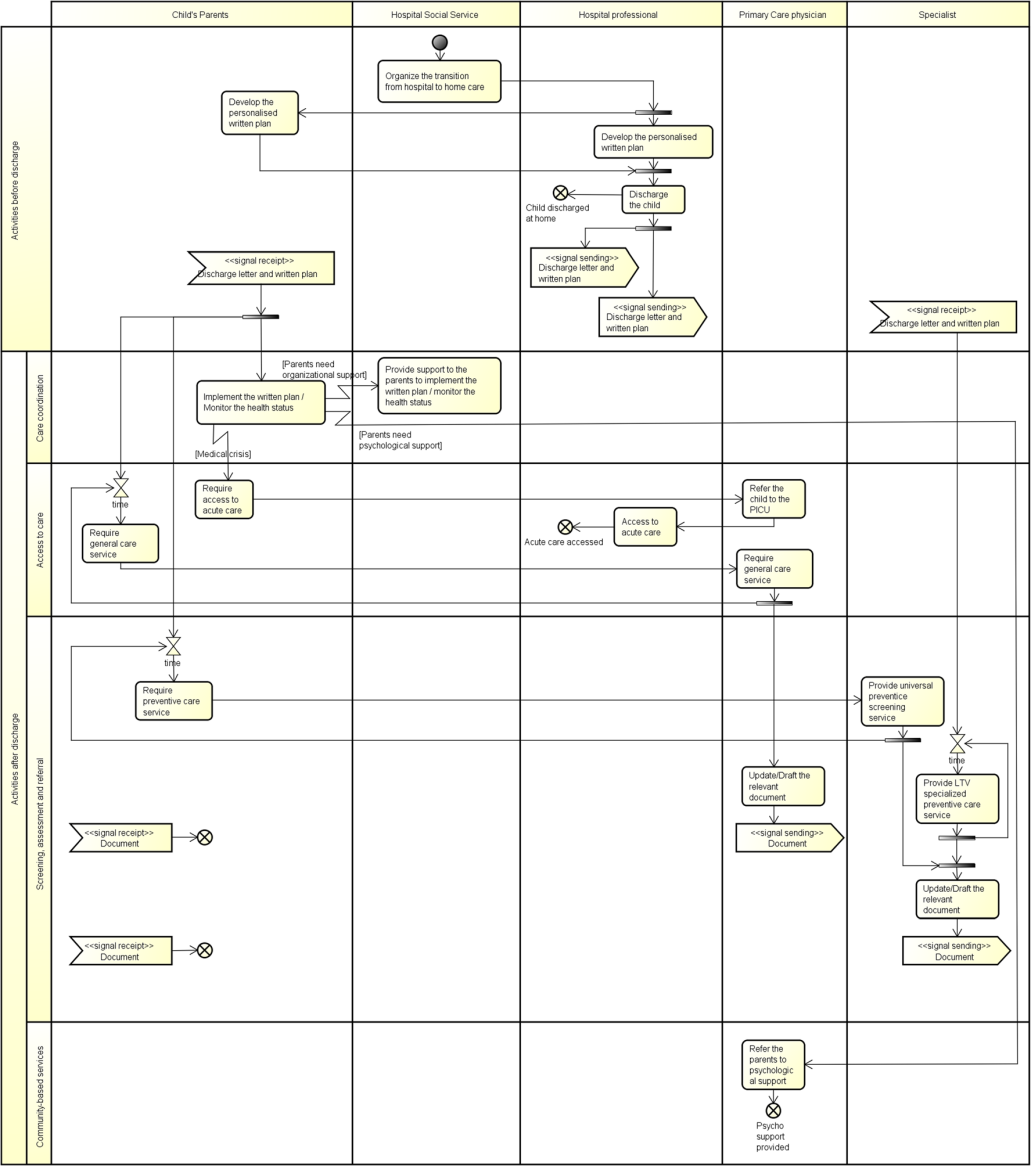
UML activity diagram: Luxembourg

From the results of the clustering analysis, two activity diagrams are shown in Figs. 4 and 5. These two diagrams represent the countries falling in the two extremes of the model reported in Fig. 2.

In each diagram, the vertical partition is used to indicate the actors that perform group of activities and messages sent/received. In this case, actors are individual professionals (e.g. discharge planning coordinator, primary care professionals, hospital specialist) or a group of professionals working in a team (e.g. primary care team, multidisciplinary team, hospital team). Moreover, horizontal partitions are used to classify the activities within the following components of the CYSCHN [16]: (1) care coordination (i.e. implementation of the written plan); (2) access to care (i.e. acute services in case of medical crisis and provision of curative services); (3) screening, assessment and referral (i.e. provision of preventive care screening and developmental checks); (4) community-based services (i.e. psychological support to parents and provision of palliative care).

#### Fully-mixed MDT: Estonia as an exemplar

In Estonia (Fig. 4), the MDT is composed of both hospital and primary care professionals. In particular, the main role within the child’s care pathway is played by the hospital MDT that organises the transition of the child from the acute hospital setting to the child’s home and develops the personalised written plan in collaboration with the parents. Before discharge, the child receives the disability card to simplify the certification of the degree of disability in connection with exercising the rights granted to disabled patients. The hospital team is also in charge of the implementation of the written plan with the provision of psychological support to the parents, the provision of acute care services in case of medical crisis and the provision of palliative care, where appropriate, after consulting with the parents. The primary care professionals that compose the MDT are involved after discharge in the provision of both preventive care and general curative services. Generally, in Estonia, these services can be provided either at the hospital or at the primary care level. These two teams communicate the results of each test though the e-health system.

#### Individual professionals: Luxembourg as an exemplar

In Luxembourg (Fig. 5), the transition of the child from the acute hospital setting to the child’s home is organised by the hospital social service, while the personalised plan is developed by the hospital health care professional in collaboration with the child’s parents. After discharge, the hospital physician shares the written plan and the discharge letter with the parents who are in charge of requiring and managing the routine preventive screening services. At the same time, the hospital physician refers the child to relevant specialists for the organisation and provision of LTV-specialised services. The documents produced during the visits are collected by the child’s parents who are also in charge of the implementation of the written plan with the support of the hospital social services, if needed and required by the parents. During the monitoring of the child’s health status, the parents can require access to acute care in case of a medical crisis through the primary care physician, who refers the child to the PICU that provides the services. The primary care professional is also involved in the provision of psychological support to the parents by referring them to the relevant psychologist.

## Conclusions and discussion

The paper describes the level of collaboration among primary and secondary professionals in place in the MOCHA countries to coordinate and provide services to children dependent on LTV. The adoption of a business process analysis allowed us to identify different patterns of collaboration that may be distributed along a continuum of integration. Moreover, the use of a standardised modelling language (UML) made it possible to represent the different organisational features in place in the MOCHA countries to manage children with LTV in a comparable way.

Within a high variety of patterns of collaboration, primary care professionals are involved in the majority of countries providing general health services, while hospital professionals continue to be a reference point also when the child is discharged at home. Moreover, the presence of a social worker within a MDT either in hospital or in primary care setting indicates in some countries a trend of including the social component to identify and fulfil child and family needs. The adoption of a personalised written plan in particular when the family is involved in its development can be considered one of the optimum features in the treatment of a child with CCNs, in particular, considering that the plan should include the identification of the procedures to be adopted for access care in case of medical crisis. The communication process between professionals that either belong to a MDT or to different levels of care is facilitated by the use of shared medical records. This is particularly important when the monitoring process after discharge involves primary care professionals together with hospital specialists.

The two examples presented in this paper highlight how a team composition influences the flow of the activities in terms of process complexity. In the first example, a MDT composed by both hospital and primary care professionals performs all required activities needed to treat and monitor the child health status, while in the second one, services are provided by single professionals, each one belonging to a specific setting. In the latter case, the parents are in charge of the implementation of the written plan organising and planning the child’s care and they contact the relevant specialist each time they require their care. They also have the burden of communicating the results of each treatment provided. The differences between the two organisational processes are clearly highlighted by the number of swim lanes that represent the professionals involved in the care pathway as well as by the number of control-flow lines between activities that represent the interactions needed to perform the process of care. The presence of hourglasses in the second diagram also emphasises the waiting times between the request for a service and its delivery.

Each of these UML features could be included within an evaluation metrics to assess for instance the straightforwardness of the process indicating a smoother pathway in the organisation of childcare and a reduced burden for the parents caring for them. This implies the identification of a selection of qualitative indicators that evaluate, in our case at macro level, bottlenecks in the process and/or facilitators of its execution, such as the use of shared ICT systems. Of course, as health evaluation measures have to be built on the basis of experts’ consensus and scientific evidence, this approach should be further scrutinised and researched, especially if combined with composite indicators, such as QALYs and DALYs. Moreover, the degree of integration of care, whose benefits are generally recognised [31–33], could be evaluated on the basis of the team composition (within a similar scale we have proposed in this paper) along with the activities performed, supporting the identification of the best solutions to achieve it.

This approach provides important indications on the efficiency in performing and organising health-related activities. Further advantages in the adoption of this methodology could be achieved, if the identified activities were associated with data, such as the number and expenditure for health professionals and waiting times, to estimate the performance of the pathway under a cost-effectiveness perspective. Moreover, this could be applied to health outcomes where selected parts of the process can be associated with indicators such as avoidable and emergency hospital admissions, readmission rate and drug consumption. This could provide an in-depth, multidimensional analysis indicating best practices in the provision of care.

## Abbreviations

CCN: Complex care need
MOCHA: Models of child health appraised
LTV: Long-term ventilation
CYSCHN: Children and youth with special health care needs
UML: Unified Modelling Language
CA: Country Agent
MDT: Multidisciplinary team
PICU: Paediatric intensive care unit

## Footnotes

1 MOCHA website available at http://www.childhealthservicemodels.eu/
